# Spatial and Temporal Trends in Pancreatic Cancer Burden Attributable to High Body Mass Index at the Global and National Levels

**DOI:** 10.1007/s44197-023-00155-8

**Published:** 2023-10-05

**Authors:** Fei Cao, Feng Li, Lei Shi, Guoyao Zhang, Lei Zhang, Tianjiang Ma, Kexun Zhang

**Affiliations:** 1 Department of Oncology, Luohe Central Hospital, People’s East Road 54, Luohe City, 462000 Henan Province China; 2 Department of General Surgery, Shaoling District People’s Hospital of Luohe, Luohe City, 462000 Henan Province China; 3 Department of Infectious Disease Control, Kunshan Centers for Disease Control and Prevention, Tongcheng South Road 567, Kunshan, 215300 Jiangsu China

**Keywords:** Pancreatic cancer, Overweight, Global burden of disease, Spatiotemporal trends

## Abstract

**Objectives:**

To examine the spatiotemporal trends in pancreatic cancer (PC) disability-adjusted life years (DALYs) and mortality attributable to high body-mass index (BMI) by age, gender, and countries from 1990 to 2019.

**Methods:**

Data were extracted from the Global Burden of Disease Study 2019 results. We presented the annual number of PC DALYs and mortality, and corresponding age-standardized rates (ASDR and ASMR), which were further stratified by age, gender, and countries. The estimated annual percentage change (EAPC) was computed to assess the longitudinal trends in ASRs.

**Results:**

In 2019, 0.7 million DALYs and 31.9 thousand deaths worldwide were caused by PC attributable to high BMI, with the largest amount reported in high-income North America, Western Europe, and East Asia. The corresponding ASDR and ASMR were highest in females and in high SDI regions, while quite varied across countries. The global EAPC in ASDR and ASMR was 1.45 (95% uncertainty interval [UI]: 1.40, 1.50) and 1.44 (95% UI: 1.39, 1.49), respectively. Almost all involved countries demonstrated significant uptrends in ASRs from 1990 to 2019.

**Conclusions:**

More productive efforts to reduce the impact of modifiable risk factors, such as overweight, should be undertaken, and thus effectively curb the rise of PC burden.

**Supplementary Information:**

The online version contains supplementary material available at 10.1007/s44197-023-00155-8.

## Introduction

Pancreatic cancer (PC) was considered as one of the most aggressive tumors with poor prognosis. It was estimated to be the fifth cause of global deaths and the eighth of global disability-adjusted life years (DALYs) in 2019 [1]. That year, there were approximately 531 thousand deaths and 11.5 million absolute DALYs causing by PC worldwide [1]. A recent review indicated that modifiable exposures, including cigarette smoking, diabetes mellitus, obesity, and alcohol use, enhanced the risk of PC, and played a pivotal role in the upward trend over the past few decades [2]. In addition, a majority of patients with this malignant disease tended to be found at an advanced stage and had extremely low 5-year survival rate. Hence, it may be of practical significance to formulate prevention and management strategies to curb its rise.

Overweight and obesity have achieved epidemic proportions and are acknowledged as modifiable risk factors for many diseases, for instance, cardiovascular diseases and several cancers [3, 4]. The global DALYs and deaths owing to high body-mass index (BMI, BMI ≥ 25 kg/m^2^) had a twofold sharp rise from 1990 to 2017 [3]. High BMI was reported to be one of the merely three risk factors contributing to more than 1% of DALYs, and increased in all socio-demographic index (SDI) quantiles during the past decades [5], especially in low- and low-middle SDI regions. Furthermore, in 2019, high BMI occupied the top four positions as a risk factor for attributable DALYs in the entire population exceeding the age of 25 [5]. Higher BMI has been recognized to be one of the pivotal risk factors for pancreatic cancer, but its contribution varied globally [2].

Description of the epidemiologic features of PC owing to high BMI at the global and national scales was rare, while this information had scientific implications for policy making. Our study purposed to examine spatiotemporal trends in PC DALYs and deaths attributable to high BMI by age, gender, and regions between 1990 and 2019.

## Materials and Methods

### Data Sources

In this study, data concerning the annual number of pancreatic cancer DALYs and deaths, and the age-standardized rate of DALYs (ASDR) and deaths (ASMR) owing to high BMI from 1990 to 2019 were extracted from the Global Burden of Disease Study (GBD) 2019 results. For the sake of clarity, research data stratified by gender, age, region, and country. Specifically, the GBD database covered 204 countries and territories, and were further divided into 21 geographic locations [6]. According to the SDI, the world was grouped into five regions, comprising low, low–middle, middle, high–middle, and high SDI regions [6]. Moreover, the age was divided into three groups, namely, 15–49 age group, 50–69 age group, and more than 70 age group. We presented all the estimates with 95% uncertainty interval (UI) (derived from the 2.5th and 97.5th percentile values of the ordered 1000 draws), which was on basis of measuring error, system biases, and modelling. This method had been described elsewhere in detail [6]. BMI ≥ 25 kg/m^2^ was considered as high BMI or overweight. SDI was a composite index developing by the GBD researchers, which was recognized to link developing status with health outcomes closely. In theory, a score of 0 represented the least developed, while a score of 1 represented the most developed [7].

### Statistical Analysis

To improve comparability between regions and countries, the age standardization of DALY and mortality rate was necessary, which could eliminate the influence originated from age structure in different populations. We determined the estimated annual percentage change (EAPC) as the changing trends of ASRs over the study period. The computation of EAPC, with its 95% confidence interval, was based on two regression models as follows: ln(ASR) = α + β·*x* + ε, where *x* = calendar year; and then EAPC = 100 × (exp(β) − 1) [8]. The ASDR and ASMR were recognized to trend upward when the corresponding EAPC index and the lower bound of its 95% CI were > 0; they were thought to trend downward when the corresponding EAPC index and the upper bound of the 95% CI < 0; otherwise, the trends were considered to hold steady. Previous studies revealed that socioeconomic status (SES) may have associations with the treatment options and prognosis of patients with pancreatic cancer [9–11]. While the Human Development Index (HDI) was a good gauge for SES to some extent. We additionally sought to examine whether HDIs was related to the EAPCs at country and territory levels in 2019 by use of Pearson’s correlation analysis. The HDIs of various countries in 2019 were retrieved from the World Bank (www.worldbank.org) website. We performed all the statistical analyses using R program (R core team, Version 1.2.1335). A two-sided *P* value < 0.05 was considered statistically significant.

## Results

### Global Spatial Patterns of Pancreatic Cancer Burden and Mortality Attributable to High Body-Mass Index

In 2019, pancreatic cancer resulted in an estimated 3.7 million DALYs and 173.7 thousand deaths worldwide, of which nearly, 0.7 million DALYs and 31.9 thousand deaths were attributed to high BMI, accounting for 18.9% and 18.4%. In this section, for brevity, we referred to pancreatic cancer owing to high BMI as pancreatic cancer.

**Fig. 1:**
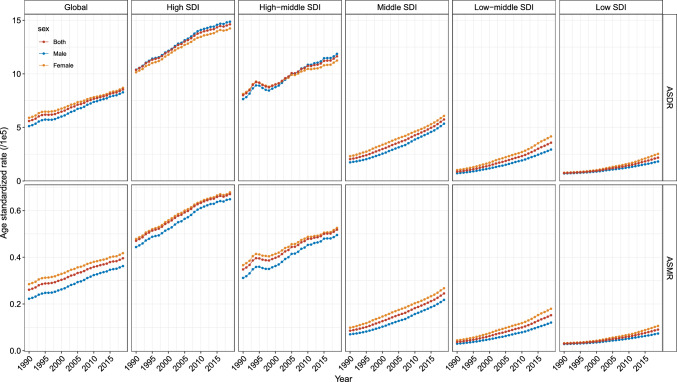
Temporal trends in age-standardized rates of pancreatic cancer disability-adjusted life years and mortality attributable to high BMI from 1990 to 2019 by gender and by sociodemographic index regions. *ASDR* age-standardized disability-adjusted life year rate, *ASMR* age-standardized mortality rate, *SDI* sociodemographic index

Among the five SDI regions, the number of pancreatic cancer DALYs in higher SDI regions was greater than that in lower SDI regions (Table 1). High SDI regions had the highest pancreatic cancer DALYs of 0.26 million, which was more than 20 times of that in low SDI regions. In parallel, the largest number of deaths was observed in high SDI regions, four times as many as in low SDI regions. Besides, as for ASRs, both ASDR and ASMR increased with higher SDI (Table 1, Fig. 1).

**Table 1:** ASDR and ASMR of PC attributable to high BMI in 1990 and 2019, and their EAPCs between 1990 and 2019

Characteristics	1990				2019				EAPC (1990–2019)	
	DALYs		Deaths		DALYs		Deaths		ASDR	ASMR
	No. × 10^3^ (95% UI)	ASDR	No. (95% UI)	ASMR	No. × 10^3^ (95% UI)	ASDR	No. (95% UI)	ASMR	No. (95% UI)	No. (95% UI)
		No. × 10^–5^ (95% UI)		No. × 10^–7^ (95% UI)		No. × 10^–5^ (95% UI)		No. × 10^–7^ (95% UI)		
Global	224.3	5.60	9693	26.09	709.4	8.54	31,921	39.56	1.45	1.44
	(80.5, 441.1)	(2.04, 11.01)	(3522, 18,997)	(9.45, 50.87)	(255.9, 1325.4)	(3.09, 15.99)	(11,964, 59,676)	(14.97, 73.88)	(1.40, 1.50)	(1.39, 1.49)
Sex										
Female	125.7	5.90	5851	28.48	380.0	8.69	18,323	41.76	1.30	1.30
	(41.3, 231.6)	(1.94, 10.87)	(1921, 10,753)	(9.34, 52.44)	(133.0, 673.6)	(3.04, 15.41)	(6478, 32,800)	(14.77, 74.77)	(1.26, 1.34)	(1.25, 1.34)
Male	98.6	5.12	3842	22.24	329.5	8.27	13,598	36.21	1.68	1.74
	(-0.6, 247.0)	(0, 12.85)	(-22, 9647)	(0, 56.12)	(-2.0, 798.0)	(0, 20.05)	(-83, 33,194)	(0, 88.92)	(1.62, 1.75)	(1.68, 1.80)
Socio-demographic index										
High	106.2	10.37	4945	47.04	256.9	14.62	12,901	67.02	1.23	1.27
	(38.2, 208.4)	(3.65, 20.40)	(1818, 9504)	(17.28, 90.60)	(92.0, 477.7)	(5.23, 27.31)	(4714, 23,834)	(24.59, 124.42)	(1.15, 1.31)	(1.20, 1.35)
High–middle	88.0	8.01	3635	34.79	238.6	11.64	10,576	51.79	1.20	1.32
	(32.0, 169.7)	(2.95, 15.43)	(1354, 6932)	(12.93, 66.12)	(85.2, 449.1)	(4.15, 21.94)	(3931, 19,664)	(19.39, 96.30)	(1.09, 1.31)	(1.23, 1.42)
Middle	22.5	2.04	834	8.57	150.4	5.74	5974	24.50	3.66	3.72
	(7.1, 47.9)	(0.64, 4.33)	(263, 1765)	(2.68, 18.28)	(55.2, 293.4)	(2.11, 11.23)	(2201, 11,678)	(8.92, 47.47)	(3.59, 3.73)	(3.64, 3.80)
Low–middle	5.6	0.87	207	3.73	51.1	3.57	2005	15.19	5.07	5.04
	(1.6, 12.9)	(0.25, 2.04)	(58, 484)	(1.02, 8.78)	(18.9, 98.7)	(1.32, 6.92)	(740, 3934)	(5.58, 29.77)	(4.99, 5.14)	(4.94, 5.13)
Low	1.9	0.74	69	3.02	12.2	2.18	449	9.03	4.01	4.07
	(0.5, 4.6)	(0.18, 1.79)	(17, 167)	(0.74, 7.42)	(4.1, 24.6)	(0.73, 4.42)	(151, 917)	(2.98, 18.42)	(3.85, 4.17)	(3.92, 4.22)
GBD region										
High-income Asia Pacific	10.3	5.02	452	22.88	23.2	5.70	1315	27.59	0.36	0.60
	(2.4, 24.8)	(1.18, 12.01)	(105, 1083)	(5.34, 55.06)	(6.1, 53.7)	(1.46, 13.15)	(335, 3019)	(7.21, 63.77)	(0.29, 0.44)	(0.53, 0.67)
Central Asia	1.8	3.65	68	14.93	8.8	11.11	334	47.99	4.78	4.98
	(0.6, 3.4)	(1.33, 6.95)	(25, 130)	(5.43, 28.09)	(3.3, 16.1)	(4.23, 20.29)	(130, 608)	(19.60, 86.27)	(4.30, 5.25)	(4.52, 5.45)
East Asia	13.5	1.41	484	5.64	110.2	5.11	4402	21.20	4.83	4.94
	(2.4, 36.5)	(0.25, 3.75)	(86, 1293)	(1.01, 15.05)	(28.1, 247.5)	(1.31, 11.49)	(1168, 9933)	(5.57, 47.75)	(4.62, 5.04)	(4.72, 5.16)
South Asia	2.8	0.46	103	1.95	35.4	2.41	1393	10.25	6.03	6.01
	(0.7, 7.0)	(0.11, 1.16)	(25, 260)	(0.46, 4.97)	(12.7, 67.9)	(0.86, 4.60)	(501, 2682)	(3.70, 19.83)	(5.88, 6.17)	(5.83, 6.19)
Southeast Asia	2.9	0.99	98	3.84	26.5	4.02	990	16.50	4.90	5.15
	(0.7, 7.1)	(0.24, 2.48)	(23, 247)	(0.92, 9.66)	(9.4, 52.9)	(1.43, 7.97)	(351, 1977)	(5.89, 33.25)	(4.82, 4.98)	(5.09, 5.22)
Australasia	2.4	10.39	110	47.00	6.9	14.67	347	68.64	1.28	1.37
	(0.9, 4.6)	(3.69, 20.00)	(40, 209)	(16.93, 89.12)	(2.6, 12.3)	(5.46, 26.32)	(133, 621)	(26.07, 122.99)	(1.21, 1.35)	(1.29, 1.44)
Caribbean	0.5	2.05	22	8.70	4.6	8.90	201	38.79	5.07	5.12
	(0.2, 1.0)	(0.77, 3.88)	(8, 42)	(3.24, 16.49)	(1.7, 8.6)	(3.32, 16.61)	(77, 375)	(14.82, 72.16)	(4.26, 5.88)	(4.30, 5.95)
Central Europe	22.3	14.87	919	62.40	45.3	22.20	2083	96.50	1.44	1.58
	(7.7, 42.2)	(5.16, 28.20)	(335, 1717)	(22.96, 116.40)	(16.6, 82.9)	(7.96, 40.98)	(792, 3741)	(36.34, 174.32)	(1.34, 1.53)	(1.49, 1.66)
Eastern Europe	34.3	12.00	1365	48.38	60.7	18.00	2593	74.80	1.14	1.34
	(13.3, 63.4)	(4.67, 22.20)	(524, 2480)	(18.63, 87.82)	(23.7, 110.6)	(6.90, 32.81)	(1037, 4647)	(29.76, 134.23)	(0.85, 1.44)	(1.07, 1.61)
Western Europe	58.2	10.42	2814	48.05	117.4	14.10	6336	67.44	1.09	1.23
	(20.8, 114.6)	(3.63, 20.70)	(1032, 5425)	(17.66, 93.20)	(42.4, 218.2)	(4.97, 26.66)	(2355, 11,733)	(24.35, 125.44)	(0.98, 1.21)	(1.12, 1.34)
Andean Latin America	0.6	2.57	21	10.44	5.6	9.88	239	43.35	4.95	5.27
	(0.2, 1.1)	(0.94, 5.06)	(7, 42)	(3.65, 21.21)	(2.2, 10.2)	(3.89, 17.84)	(96, 435)	(17.29, 78.79)	(4.30, 5.61)	(4.59, 5.97)
Central Latin America	5.7	6.44	219	27.15	23.8	9.88	995	42.67	1.24	1.31
	(2.2, 10.7)	(2.44, 12.16)	(82, 411)	(10.28, 51.00)	(9.2, 43.7)	(3.84, 18.17)	(400, 1830)	(17.20, 78.33)	(1.10, 1.37)	(1.16, 1.46)
Southern Latin America	4.7	9.98	204	44.88	14.1	17.12	671	79.62	1.68	1.81
	(1.6, 9.4)	(3.41, 20.08)	(70, 409)	(15.27, 89.37)	(5.4, 26.3)	(6.47, 31.99)	(260, 1231)	(30.68, 146.43)	(1.39, 1.97)	(1.52, 2.10)
Tropical Latin America	6.0	6.21	233	26.81	27.6	11.18	1197	50.12	2.18	2.32
	(2.1, 11.9)	(2.17, 12.35)	(81, 464)	(9.48, 53.87)	(10.6, 50.3)	(4.29, 20.31)	(467, 2160)	(19.73, 90.28)	(2.10, 2.25)	(2.25, 2.39)
North Africa and Middle East	7.5	4.06	277	16.68	52.3	11.26	2006	48.26	3.59	3.79
	(2.5, 14.7)	(1.35, 7.94)	(94, 541)	(5.71, 32.46)	(18.5, 96.9)	(4.07, 20.75)	(741, 3702)	(18.09, 87.76)	(3.44, 3.75)	(3.63, 3.96)
High-income North America	46.2	13.74	2126	60.14	120.7	20.03	5814	91.13	1.33	1.48
	(17.7, 85.2)	(5.18, 25.35)	(832, 3911)	(23.37, 110.55)	(45.7, 214.6)	(7.50, 35.50)	(2244, 10,302)	(34.99, 161.94)	(1.21, 1.44)	(1.37, 1.60)
Oceania	0.1	2.07	2	8.19	0.3	3.35	9	13.71	1.43	1.56
	(0.0, 0.1)	(0.66, 4.36)	(1, 5)	(2.60, 17.52)	(0.1, 0.5)	(1.11, 6.82)	(3, 19)	(4.58, 27.84)	(1.09, 1.76)	(1.26, 1.86)
Central Sub-Saharan Africa	0.4	1.42	13	5.77	1.5	2.44	51	9.96	1.37	1.39
	(0.1, 0.8)	(0.37, 3.19)	(3, 29)	(1.50, 13.13)	(0.5, 3.1)	(0.78, 5.28)	(16, 111)	(3.22, 21.10)	(0.79, 1.96)	(0.80, 1.98)
Eastern Sub-Saharan Africa	0.7	0.89	26	3.61	5.0	2.80	181	11.46	4.33	4.38
	(0.2, 1.8)	(0.22, 2.17)	(7, 64)	(0.89, 8.89)	(1.8, 10.1)	(1.01, 5.67)	(64, 365)	(4.01, 23.00)	(4.07, 4.59)	(4.12, 4.65)
Southern Sub-Saharan Africa	2.0	7.10	79	30.50	7.3	12.58	300	56.98	1.98	2.16
	(0.8, 3.7)	(2.85, 13.01)	(32, 144)	(12.07, 55.70)	(3.0, 12.8)	(5.20, 21.90)	(126, 519)	(23.72, 98.37)	(1.71, 2.25)	(1.89, 2.42)
Western Sub-Saharan Africa	1.5	1.55	55	6.40	12.3	6.12	465	26.25	4.79	4.95
	(0.5, 3.2)	(0.48, 3.39)	(17, 120)	(1.98, 14.11)	(5.0, 23.5)	(2.46, 11.60)	(186, 883)	(10.41, 50.15)	(4.70, 4.88)	(4.87, 5.02)

*ASDR* age-standardized disability-adjusted life year rate, *ASMR* age-standardized mortality rate, *DALY* disability-adjusted life year, *EAPC* estimated annual percentage change, *UI* uncertainty interval, *GBD* global burden of disease study, *No.* number, *PC* pancreatic cancer, *BMI* body-mass index

In terms of geographical location, the largest amount of pancreatic cancer DALYs and deaths were reported in three areas, including high-income North America, Western Europe, and East Asia (Table 1) which together accounted for approximately half of the world total (DALYs: 49.1%, deaths: 51.9%). On the contrary, the Oceania reported the fewest pancreatic cancer DALYs, followed by Central Sub-Saharan Africa and then Caribbean. While the fewest deaths occurred in Oceania, Central Sub-Saharan Africa, and Eastern Sub-Saharan Africa in turn. With regard to ASRs, Central Europe, high-income North America, and Eastern Europe had the highest ASDR, while the highest ASMR was found in Central Europe, high-income North America, and Southern Latin America. On the other hand, the lowest ASDR and ASMR were observed in South Asia, Central Sub-Saharan Africa, and Eastern Sub-Saharan Africa.

At the national level, there was a prominent discrepancy in pancreatic cancer ASDR and ASMR reported by various countries in 2019 (Table S1, Fig. 4A). United Arab Emirates (47.46/100000 person-years, 95% UI 7.57, 106.65), together with Greenland (35.50/100000 person-years, 95% UI 12.46, 67.50) and Monaco (31.60/100000 person-years, 95% UI 11.57, 57.31) were the three countries and territories with the highest ASDR, exceeding 30/100000 person-years. While seven countries and territories reported ASDR < 0.05/100000 person-years, with the lowest being Somalia (0.02/100000 person-years, 95% UI 0.00, 0.06). According to statistics, 102 countries and territories had an ASDR beyond 10/100000 person-years, accounting for half of the total. Likewise, United Arab Emirates (1.98/100000 person-years, 95% UI 0.34, 4.33), Greenland (1.55/100000 person-years, 95% UI 0.55, 2.92), and Monaco (1.52/100000 person-years, 95% UI 0.55, 2.77) reported the largest ASMR, and Somalia had the lowest ASMR (0.02/100000 person-years, 95% UI 0.00, 0.06) worldwide. It was noteworthy ten countries and territories, including Hungary, Czech Republic, Virgin Islands, etc. had an ASMR more than 1.0/100000 person-years.

### Global Pancreatic Cancer Burden and Mortality Attributable to High Body-Mass Index by Gender and Age

On a global scale in 2019, compared to males, greater amount of pancreatic cancer DALYs and deaths occurred in females. The recorded numbers of DALYs and deaths were 1.15- and 1.35-times higher in females than in males, respectively. Meanwhile, females had comparatively higher ASDR and ASMR.

**Fig. 2:**
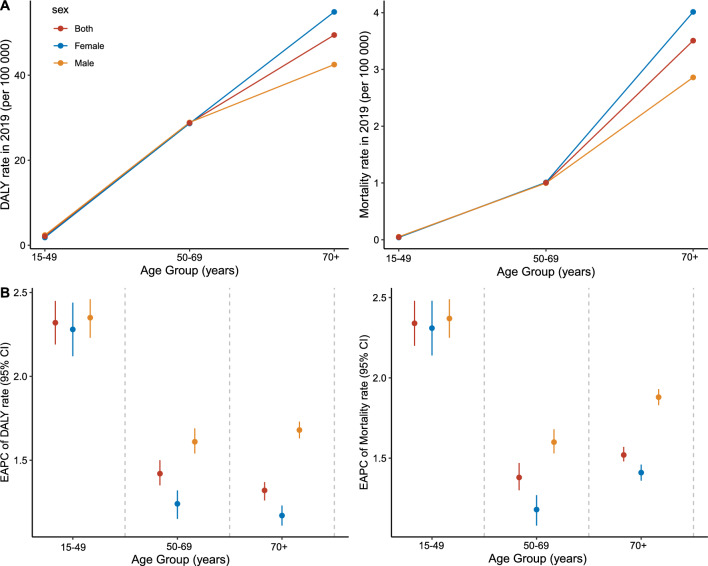
Global age-specific rates of pancreatic cancer DALYs and mortality attributable to high BMI by gender in 2019, and their EAPC rates between 1990 and 2019. **A** Global age-specific rates of pancreatic cancer DALYs and mortality by gender in 2019; **B** global EAPC rates in pancreatic cancer DALYs and mortality by age group and gender between 1990 and 2019. *DALYs* disability-adjusted life years, *EAPC* estimated annual percentage change

As illustrated in Fig. 2A, the rates of pancreatic cancer DALY and death showed similar age-specific increasing patterns. In addition, the ASDR and ASMR were nearly identical for men and women in the 15–49 and 50–69 age groups, but were relatively higher in women over 70 years.

### Temporal Trends in Pancreatic Cancer Burden and Mortality Attributable to High Body-Mass Index from 1990 to 2019

Over the past three decades, the amount of pancreatic cancer DALYs and deaths were estimated to raise by 216.3% and 229.3%, respectively (Table 1). The ASDR and ASMR also increased synchronously during this period, regardless of gender and age group. However, in specific, the increase was more pronounced in males, and the most remarkable rise was found in the 15–49 age group (Fig. 2B).

**Fig. 3:**
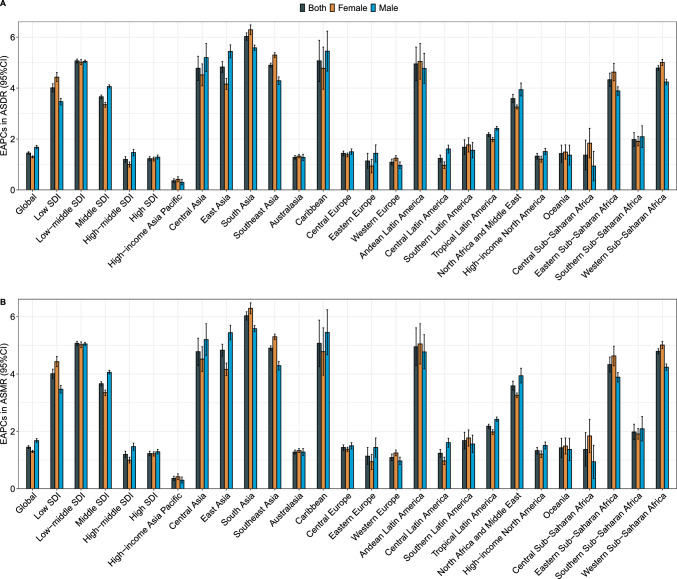
EAPCs in age-standardized rates of pancreatic cancer DALYs (**A**) and mortality (**B**) attributable to high BMI for both sexes and by gender, by sociodemographic index, and by geographic locations from 1990 to 2019. *EAPC* estimated annual percentage change, *ASDR* age-standardized disability-adjusted life year rate, *ASMR* age-standardized mortality rate, *SDI* sociodemographic index

All five SDI regions demonstrated a rise trend in ASDR and ASMR from 1990 to 2019, regardless of gender (Table 1, Fig. 3). The most evident increase was detected in the low–middle SDI regions, and then were the low and the middle SDI regions. High- and high-middle SDI regions had comparatively low increase. Among 23 geographic locations, South Asia ranked first in terms of ASDR growing trend during the 30-year study period, followed by Caribbean and Andean Latin America (Fig. 3). Furthermore, South Asia, Andean Latin America, and Southeast Asia were the three locations with the highest increases in ASMR.

**Fig. 4:**
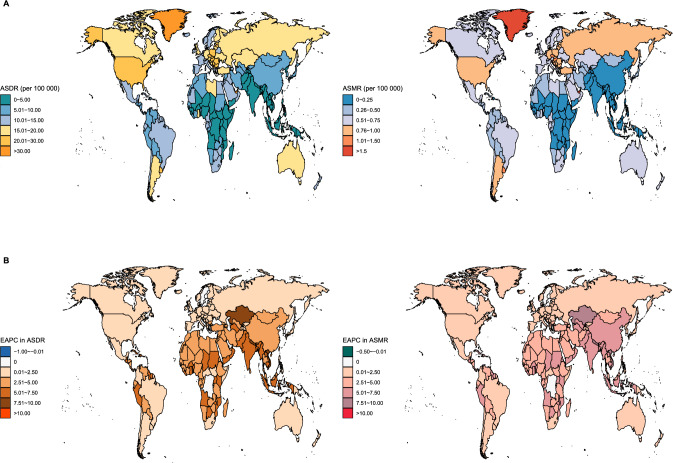
Global distribution of age-standardized rates of pancreatic cancer DALYs and mortality attributable to high BMI in 2019, and their EAPCs between 1990 and 2019. **A** Global distribution of age-standardized rates of pancreatic cancer DALYs and mortality in 2019; **B** global distribution of EAPCs in age-standardized rates of pancreatic cancer DALYs and mortality between 1990 and 2019. *ASDR* age-standardized disability-adjusted life year rate, *ASMR* age-standardized mortality rate, *EAPC* estimated annual percentage change, *DALYs* disability-adjusted life years

At the country level, almost all involved countries and territories expressed a rise trend in both ASDR and ASMR from 1990 to 2019. Of all, the highest increase of ASR was detected in Equatorial Guinea (EAPC in ASDR: 12.29, 95% UI 11.47, 13.12; EAPC in ASMR: 12.82, 95% UI 11.98, 13.67), followed by Cabo Verde (EAPC in ASDR: 9.22, 95% UI 7.81, 10.65; EAPC in ASMR: 9.53, 95% UI 8.10, 10.99). By contrast, there were only one country manifesting a significant decline trend in ASR of DALY and death, namely, Samoa (EAPC in ASDR: − 0.51, 95% UI − 0.86, − 0.16; EAPC in ASMR: − 0.42, 95% UI − 0.76, − 0.08). In addition, three countries, including Sweden, Democratic Republic of the Congo, and Kiribati, presented statistically non-significant increase in both ASDR and ASMR, suggesting that the pancreatic cancer burden stabilized during 30-year period. In general terms, a total of 47 countries and territories recorded an EAPC >  = 5.0 in ASDR, while 51 countries and territories recorded an EAPC >  = 5.0 in ASMR (Fig. 4).

**Fig. 5:**
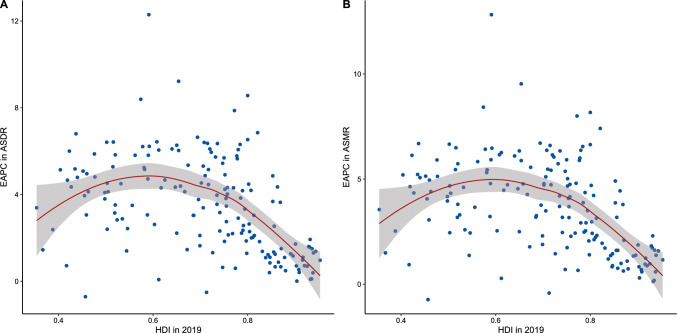
Correlations between EAPCs in ASDR (**A**) and ASMR (**B**) and the human development index in 2019. *ASDR* age-standardized disability-adjusted life year rate, *ASMR* age-standardized mortality rate, *EAPC* estimated annual percentage change, *HDI* human development index

We also evaluated the correlations between EAPC in ASRs and HDI in 2019 (Fig. 5). EAPC in ASDR and HDI illustrated an inverted U-shape association, and the peak appeared at around 0.6 of HDI (ρ = − 0.41, *P* < 0.001). So did EAPC in ASMR (ρ = − 0.43, *P* < 0.001).

## Discussion

On basis of the GBD 2019 database, we comprehensively evaluated the spatial pattern and temporal trends of pancreatic cancer owing to high BMI. In general, the ASRs of pancreatic cancer related to overweight were higher in females, in older age group, and in high SDI regions. During the recent three decades, the global burden of high BMI-related pancreatic cancer has been in an upward trend, with the exception of four countries where it declined or remained stable. The disease burden indicated a complex invert U-shape association with the development level.

The growing problem of obesity has been widely concerned in recent years. Evidence from previous epidemiologic studies suggested that obesity was associated with around 1.5-fold elevated risk of developing pancreatic cancer [12–14]. The underlying etiological mechanisms regarding the influence of excess weight on pancreatic cancer were not fully understood. A literature found that pancreatic fatty infiltration, especially in intralobular location, was significantly associated with the presence and number of pancreatic intraepithelial neoplasia, independently of age and diabetes. This suggested that obesity accompanied by excess visceral fat was likely to promote the pancreatic oncogenesis. Alternatively, chronic inflammation, immune cell infiltration and tumor-associated neutrophils recruitment mediated obesity-induced tumor growth [15, 16].

We observed that the disease burden of pancreatic cancer owing to high BMI was heavier in females than in males. Nevertheless, the majority of the literature provided almost consistent evidence of higher morbidity and mortality of pancreatic adenocarcinoma among males than among females [17]. This seeming paradoxical phenomenon may be partially explained by the discrepancy in the prevalence rate of overweight and obesity in older adults. Females had significantly higher rate than males [4, 18]. Meanwhile, patients tended to be diagnosed later in life, with a median age of roughly 70 years [19, 20]. Notably, the increasing trend toward ASDR and ASMR was more prominent in males from 1990 to 2019. It may remind that gender disproportions in exposure to known or unknown risk factors may also play a part, for example, tobacco use and alcohol intake, which might have an interaction effect with excess weight on pancreatic cancer. In addition, both static data for 2019 exhibited a rise trend with age, and dynamic EAPC data from 1990 to 2019 expressed an upward trend in all three age groups. Aging was clearly the most remarkable risk factor for PC. Along with life expectancy in the general population prolonged, this alteration trend may contribute to the gradually increasing prevalence and incidence of PC in the elderly to some degree [21]. Hence, comprehensive and multifaceted primary preventive measures against modifiable risk factors were warranted to reduce the global burden of this disease.

There existed wide variations of high BMI-associated PC burden across regions and countries universally. In general, high-income countries carried heavier ASRs of disease burden than low-income countries. Overall rates in high SDI regions were nearly three times than in middle SDI regions, and seven times than in low SDI regions. This result was partly coincident with two previous meta-analysis of several prospective observational studies. The authors noted that individuals with per 5 kg/m^2^ increase in BMI was positively associated with an elevated risk of PC, with the significant associations observed in North American population and European and Australian population, while not in Asia–Pacific population [22, 23]. Differences in socioeconomic development seemed to be one reason, since the prevalence of high BMI broadly raised with the increase of SDI level [4]. A certain level of national wealth could serve as environmental prerequisite for promoting the development of obesity. Innovations and improvements in technology have also boosted food production, making consumption-based food energy intake more accessible and affordable. This boom inevitably driven up adult obesity rates [24]. From another point of view, shifts in age composition, advanced diagnostic techniques, and relatively extensive surveillance system probably accounted for the disparities in the incidence and prevalence of pancreatic cancer under various economic settings [9, 25]. However, the EAPCs in ASDR and ASMR of PC attributable to high BMI expressed inverted U-shape associations with HDI, which was indicative of a complex relation. The potential explanation may be that high BMI appeared an invert U-shape relation with socioeconomic status [5].

Admitting that the relevant imaging techniques, therapeutic schedules, and surgical procedures have witnessed great progress, the prognosis of pancreatic disease improved rarely [26]. Combined with the strong impact of living and environmental risk factors, except for Samoa, almost all other countries carried uptrend in the ASRs of high BMI-related pancreatic cancer during the study period. Among them, China was a large developing country, which owned relatively low ASDR and ASMR compared to the global average in recent 2019. Nonetheless, what is worrying is the speed of change that China carried an EAPC of nearly 5.0 in ASDR and ASMR, far above the world level. A published review manifested that the health burden of pancreatic cancer was regionally distributed in China, with a heavier burden in regions with a higher level of urbanization [27]. Similarly, in terms of overweight and obesity in China, many factors, comprising economic status, sociocultural background, and dietary preferences, contributed to regional variations in prevalence rate [28]. Therefore, more concrete evidence was needed to gain insight into how these factors affected individually or collectively to the overweight-related pancreatic cancer. Only then, through appropriate and effective public health policies, will it be possible to reduce the global burden of disease in the future.

Our study provided systematical assessment on the spatial and temporary trajectory of pancreatic cancer attributable to high BMI worldwide. However, there remained several limitations should be acknowledged. First, although we used the latest database covering 204 countries and regions, and the data from certain less developed countries may be spotty. Specialist medical practitioners and diagnostic equipment for identifying pancreatic cancer patients were likely to be insufficient. Second, the cutoff value adopted to distinguish high-BMI people may not be appropriate for some subpopulations, for example, the Asian and individuals with high risk factors, which possibly induce part of error estimation.

## Conclusion

In summary, we assessed the spatiotemporal distribution in pancreatic cancer burden and mortality attributable to high BMI over the past three decades. The disease burden attributable to overweight kept prominent in females, in older population, and in high-income locations. Unfortunately, almost all countries saw an increase in the burden from 1990 to 2019. For this almost completely fatal cancer, more productive efforts and public health policies to reduce the impact of modifiable risk factors, such as overweight, should be undertaken, and thus effectively curb the current rise in pancreatic cancer.

### Supplementary Information

Below is the link to the electronic supplementary material.

Supplementary file1 (XLS 72 KB)

## Data Availability

The data that support the findings of this study are openly available in Global Health Data.

## Abbreviations

PC: Pancreatic cancer
DALYs: Disability-adjusted life years
BMI: Body-mass index
SDI: Socio-demographic index
ASDR: Age-standardized rate of DALYs
ASMR: Age-standardized rate of deaths
GBD: The Global Burden of Disease Study
95% UI: 95% Uncertainty interval
EAPC: The estimated annual percentage change
SES: Socioeconomic status
HDI: Human development index
